# MicroRNAs Associated with Shoulder Tendon Matrisome Disorganization in Glenohumeral Arthritis

**DOI:** 10.1371/journal.pone.0168077

**Published:** 2016-12-16

**Authors:** Finosh G. Thankam, Chandra S. Boosani, Matthew F. Dilisio, Nicholas E. Dietz, Devendra K. Agrawal

**Affiliations:** 1 Department of Clinical & Translational Science, Creighton University School of Medicine, Omaha, Nebraska, United States of America; 2 Department of Orthopedic Surgery, Creighton University School of Medicine, Omaha, Nebraska, United States of America; 3 Department of Pathology, Creighton University School of Medicine, Omaha, Nebraska, United States of America; Virginia Commonwealth University Medical Center, UNITED STATES

## Abstract

The extracellular matrix (ECM) provides core support which is essential for the cell and tissue architectural development. The role of ECM in many pathological conditions has been well established and ECM-related abnormalities leading to serious consequences have been identified. Though much has been explored in regards to the role of ECM in soft tissue associated pathologies, very little is known about its role in inflammatory disorders in tendon. In this study, we performed microRNA (miRNA) expression analysis in the long head of the human shoulder biceps tendon to identify key genes whose expression was altered during inflammation in patients with glenohumeral arthritis. We identified differential regulation of matrix metalloproteinases (MMPs) that could be critical in collagen type replacement during tendinopathy. The miRNA profiling showed consistent results between the groups and revealed significant changes in the expression of seven different miRNAs in the inflamed tendons. Interestingly, all of these seven miRNAs were previously reported to have either a direct or indirect role in regulating the ECM organization in other pathological disorders. In addition, these miRNAs were also found to alter the expression levels of MMPs, which are the key matrix degrading enzymes associated with ECM-related abnormalities and pathologies. To our knowledge, this is the first report which identifies specific miRNAs associated with inflammation and the matrix reorganization in the tendons. Furthermore, the findings also support the potential role of these miRNAs in altering the collagen type ratio in the tendons during inflammation which is accompanied with differential expression of MMPs.

## Introduction

Tendinopathy of the shoulder is one of the most common causes of musculoskeletal pain and dysfunction. Tendon failure in the shoulder can lead to secondary arthritis [1]. Despite the multifactorial etiology (ageing, genetics, clinical conditions like hypercholesterolemia, habitual factors like smoking and exercise), the histomorphological alterations of the tendon in the shoulder, such as the rotator cuff and biceps tendon, are uncommon. The major histopathological changes associated with tendinopathy and rotator cuff injury (RCI) include the thinning and disorganization of the collagen fibers, fatty infiltration, calcification, apoptosis and tissue necrosis. Being highly collagenous (85% of dry weight constitutes collagen 1), tendons can effectively transform the mechanical stimuli into biochemical signals to perform specific functions. Thus, the composition, constituents and thickness of tendon extracellular matrix (ECM) are crucial for its proper functioning [2].

The actual biochemical mechanisms behind the ECM disorganization of tendinopathy are unknown. It was reported that the collagen type 1 of the matrix and collagen type 2 at the fibrocartilage junctions (which are responsible for mechanical strength) were switched to type 3 after RCI. Such change in collagen composition limits the capacity of tendon to withstand the compression stress that leads to tear [3]. A key reason for the decreased collagen 1 content is due to its active degradation by matrix metalloproteinases (MMPs) whose matrix degrading activities progressively weaken the ECM. MMP1, MMP8 and MMP13 have been classified as collagenases because of their substrate specificity towards collagen type 1, whereas MMP2 and MMP8 are grouped as gelatinases which further degrades the digestion products of collagenases. During matrix reorganization, the function of these MMPs is mainly regulated by tissue inhibitors of matrix metalloproteinases (TIMPs). The balance between MMPs and TIMPs is necessary for maintaining a normal mechanical and biochemical homeostasis of rotator cuff tendons [4].

As tendinopathy is tightly associated with inflammation and pain, the pro-inflammatory cytokines, including IL-1, IL-4, IL-6, IL-10, and TNF-α, and the pain mediators/neurotransmitters like substance P (SP) can modulate the expression of MMPs and TIMPs [5, 6]. Upregulation of MMP9 along with the inflammatory cytokines initiates a catabolic environment which aggravates the injury and delays the healing responses [7, 8]. Treatment with MMP inhibitors like doxycycline and bisphosphonates would strengthen the tendons and accelerate the repair process [9]. This suggests that MMP9 upregulation in tendon disorders results in decreased collagen type 1 with concomitant increase in collagen type 3 resulting in the sustenance of ECM disorganization. The molecular events and biochemical mechanisms underlying the hyperactivity of MMP9 in RCI patients are yet to be explored.

The expression of MMP9 is regulated by transcription factors such as AP-1 and NF-κβ as they bind to specific sites of the MMP9 promoter. The cellular stimulus which activates MEK-ERK and/or PI3K-Akt signaling activates AP-1 and NF-κβ to trigger MMP9 expression [10]. Moreover, p38 MAPK and JNK were also found to upregulate MMP9 expression [10]. Apart from these, the epigenetic regulation of MMPs, mediated by miRNAs, has gained interest in several pathological conditions such as in cancer and cardiovascular diseases. Post-translational inhibition of MMP9 enhances the tissue remodeling process and thereby facilitating the healing response [11, 12]. The miRNAs miR-491-5p, miR-125b, miR-218, miR-224, and miR-338-3p, were reported to regulate MMP9 in various cell types [11]. However, very little is known about specific miRNAs that are involved in the regulation of MMP9 and other MMPs in the tendons of rotator cuff.

The focus of the present study is to identify and evaluate the key miRNAs associated with the regulation of MMP2, MMP9, collagen type 1 and collagen type 3 in the long head of the biceps tendon in patients with and without glenohumeral arthritis. The assessment of a potential crosstalk between MMP2 and MMP9 in regulating the expression of collagen type 1 and collagen type 3, with other biochemical pathways and associated genes was explored using NetworkAnalyst program. The results from the miRNA microarray analysis were further correlated with the expression of MMPs and other potential pathway mediators associated with tendon ECM.

## Results

### Increased ECM disorganization in shoulder tendons with arthritis compared to non-arthritis group

**Fig 1:**
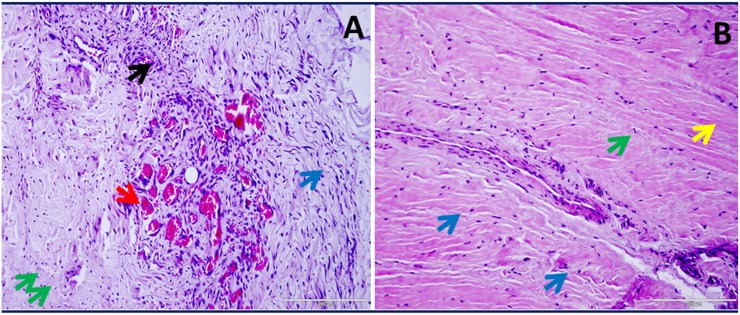
H&E staining in shoulder tendon. (A) Group 1 patient with tendinopathy as well as glenohumeral arthritis (representative of 4 individual subjects in Group 1) and (B) Group 2 patient with tendinopathy but without glenohumeral arthritis (representative of 4 individual subjects in Group 2). The green arrows show tendon cells, red arrows point blood vessels indicating angiogenesis, blue arrows indicate ECM disorganization, black arrows show inflammation and yellow arrow mark normal ECM with dense collagen deposition. The figures were taken in 400x magnification.

**Fig 2:**
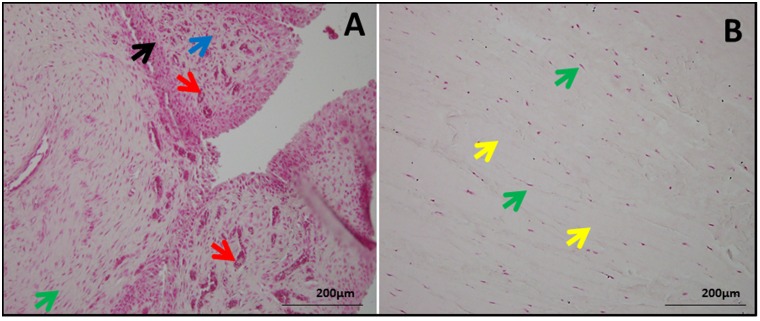
NFR staining in shoulder tendon showing the morphology and orientation. (A) Group 1 patient (representative of 4 individual subjects in Group 1) with tendinopathy as well as glenohumeral arthritis and (B) Group 2 patient (representative of 4 individual subjects in Group 2) with tendinopathy but without glenohumeral arthritis. The green arrows show tendon cells, red arrows point blood vessels indicating angiogenesis, blue arrows indicate ECM disorganization, black arrows show inflammation and yellow arrow mark normal ECM. The figures were taken in 400x magnification.

H&E (Fig 1) and nuclear fast red (NFR) (Fig 2) staining showed severe pathological features indicating ECM disorganization in the tendons of Group 1 patients when compared to Group 2 patients. The tendon cells were characterized by their elongated and less dense nuclei surrounding intact ECM which was evident in both groups. The inflammation and ECM disorganization were maximal in Group 1 tendon tissues (Figs 1A and 2A) whereas in Group 2 tissues the ECM was minimally disorganized with most of the tissue section having dense and unaltered ECM (Figs 1B and 2B). The cellular density was higher in Group 1 which can be correlated to inflammatory responses; the increased cellularity results from the infiltration of inflammatory cells especially CD16+ neutrophils and CD68+ macrophages. We have recently reported the increased density of inflammatory cells (CD16+ neutrophils and CD68+ macrophages) in the tendon tissue of patients with shoulder dysfunction as well as glenohumeral arthritis. Interestingly, the inflammatory cells were completely absent in the tendons of RCI patients without glenohumeral arthritis [13]. Also, the Group 1 tendons displayed greater vasculature resulting from extensive neoangiogenesis as a result of repair responses (Figs 1A and 2A). Group 2 patients, on the other hand, exhibited comparatively intact ECM with minimal disorganization as well as no classical signs of inflammation (Figs 1B and 2B).

**Fig 3:**
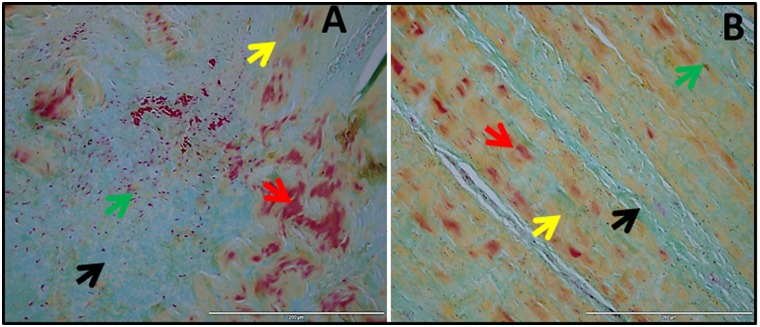
Movat pentachrome staining of shoulder biceps tendons. (A) Group 1 patient (representative of 4 individual subjects in Group 1) with tendinopathy as well as glenohumeral arthritis and (B) Group 2 patient (representative of 4 individual subjects in Group 2) with tendinopathy but without glenohumeral arthritis. The red arrows indicate fibrosis and muscle fibers, yellow arrows show collagen fibers, green arrows mark tenocytes and black arrows show mucin deposits.

Similarly, Movat pentachrome staining (Fig 3) confirmed the prominent ECM disorganization in Group 1 which was evident from the disorganized collagen. Angiogenesis was also higher in this group as confirmed with H & E and NFR staining. Movat pentachrome staining displayed a massive disorganization of ECM which was observed as disorganized collagen fibers and enhanced mucin deposition in Group 1 (Fig 3A). The muscle fiber infiltration was also vivid in Group 1 indicating the replacement of tendon tissues by cell phenotypes similar to that of myocytes (Fig 3A). But, our previous study established that the tenocytes (tenomodulin and scleraxis positive cells) were responsible for the ECM disorganization in the tendon tissues of patients with shoulder tendinopathies [13]. ECM disorganization was less severe in Group 2 (Fig 3B) when compared to Group 1 (Fig 3A) tissues indicating that inflammation associated arthritis results in severe ECM damage.

### Depletion of collagen type 1 and enhancement of collagen type 3 in shoulder tendons with arthritis compared to non-arthritis group

**Fig 4:**
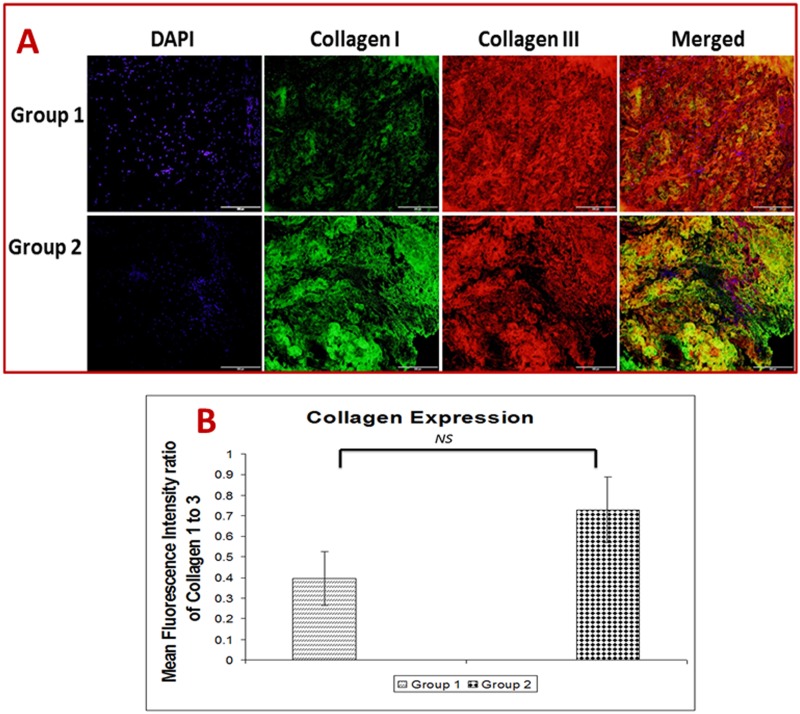
(A) Immunofluorescence analysis for the expression of collagen type 1 and type 3 in shoulder tendon. The images show an increased collagen type 3 than collagen type 1 expression in Group 1 (representative of 4 individual subjects in Group 1) and Group 2 (representative of 4 individual subjects in Group 2). (B) The ratio of collagen type 1 to type 3. The ratio was calculated using mean fluorescence intensity by ImageJ software from collagen type 1 and type 3 immunofluorescence data. The average intensities of four images taken from different sections of each patients from Group 1 (n = 4) and Group 2 (n = 4) were used for calculation of mean fluorescence intensity. The ratio of collagen type 1 to type 3 is displayed as mean ± SEM of fluorescence intensity (*NS—non-significant*)

The differential expression of collagen subtypes, collagen type 1 and type 3, were determined by dual immunostaining (Fig 4A). The amount of collagen present was quantified using ImageJ software in terms of fluorescence intensity. Fluorescence intensities were measured to estimate the ratio of collagen 1 to collagen 3, which was expressed as mean fluorescence intensity (MFI) (Fig 4B). Depletion of the collagen type 1 to type 3 was observed in both Group 1 and Group 2, but predominantly in Group 1 (Fig 4A and 4B). The ratio was found to be 0.4 MFI in Group 1 while 0.73 in Group 2. The results showed a considerable difference in the collagen ratio reflecting the extent of tissue damage and inflammation.

### Disorganized ECM altered miRNA expression in shoulder tendons

**Fig 5:**
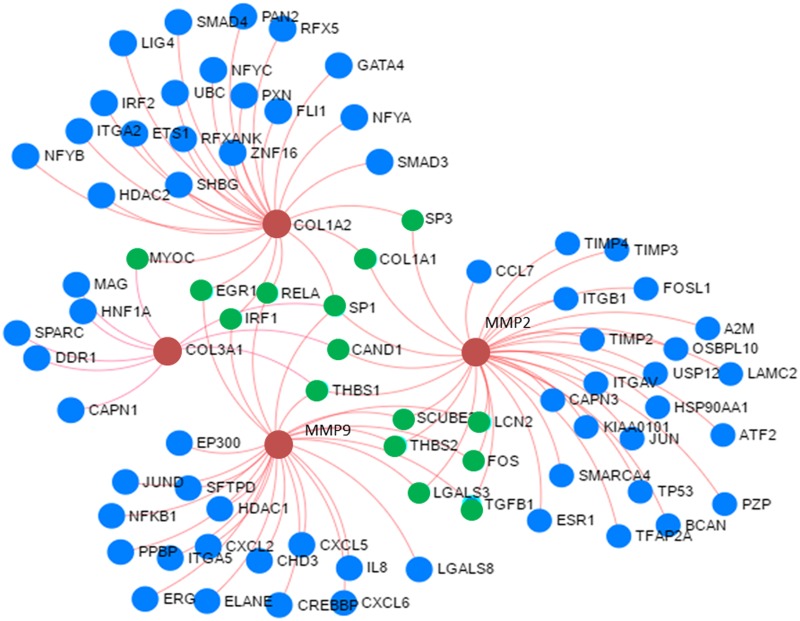
Determination of genes associated with ECM disorganization by NetworkAnalyst program. COL1A2, COL3A1, MMP9 and MMP2 were given as the input genes. The red buttons shows the input genes, green buttons represent interconnecting genes and blue buttons indicate associated genes. The output genes symbols and names/abbreviation are presented by NetworkAnalyst program based on standard Official Gene Symbol system.

The miRNA array results showed significant differences in the miRNA levels between the two groups. The 4482 miRNAs were found to be altered between the patients with and without glenohumeral arthritis where 2355 miRNAs were downregulated and 2127 miRNAs were upregulated. The relative fold change in the miRNA expression ranged from -71.26 to +5.57. The miRNAs that showed a fold change between -3 to -71.26 (197 miRNAs) and +2 to +5.57 (39 miRNAs) were screened for further analysis to identify specific gene regulations using NetworkAnalyst software based on COL1A2, COL3A1, MMP9 and MMP2 as input genes. The data obtained through NetworkAnalyst showed the involvement/interactions of 81 unique genes regulating 100 pathways which are specifically associated with COL1A2, COL3A1, MMP9 and MMP2 expression (Fig 5, Table 1, S1 Table). The 1001 miRNAs (identified from our microarray data) were found to be associated with the regulation of these 81 genes which can have implications in matrisome disorganization with respect to the glenohumeral arthritis (S2 Table). Among them 21 miRNA were found to target more than 10 targets genes which were presumed to regulate 247 genes across different pathways (Table 2). These results were further screened by increasing the lower cut off limit to above -9 fold change which included one upregulated and six downregulated miRNAs, and these 7 unique miRNAs were projected to regulate 87 genes as shown in Table 3.

**Table 1:** Genes associated with COL1A2, COL3A1, MMP9 and MMP2 as determined by NetworkAnalyst. The NetworkAnalyst program data reveals the genes interconnected with our genes of interest (COL1A2, COL3A1, MMP9 and MMP2) based on the published data base and the gene symbols are based on official gene symbol.

MMP2	LAMC2	HDAC1	USP12
MMP9	CCL7	ITGAV	OSBPL10
COL1A2	IRF2	ZNF16	SMARCA4
COL3A1	TFAP2A	CAPN3	ERG
SP1	NFYB	HSP90AA1	ELANE
THBS1	NFYA	ITGA2	MAG
SP3	NFYC	NFKB1	TIMP4
COL1A1	ATF2	CHD3	CXCL5
IRF1	FOSL1	IL8	TIMP3
RELA	JUN	CXCL2	A2M
EGR1	ESR1	PPBP	PZP
MYOC	ETS1	CXCL6	CAPN1
CAND1	FLI1	SPARC	SHBG
LCN2	RFX5	GATA4	HNF1A
THBS2	HDAC2	SMAD4	ITGA5
TGFB1	RFXANK	SMAD3	ITGB1
FOS	LGALS8	TP53	KIAA0101
LGALS3	BCAN	JUND	UBC
SCUBE3	PXN	CREBBP	LIG4
TIMP2	SFTPD	EP300	PAN2
			DDR1

**Table 2:** Highly altered miRNAs associated with the genes of interest (COL1A2, COL3A1, MMP9 and MMP2) as determined by miRNA array of Group 1 (n = 4) vs Group 2 (n = 4) of shoulder tendons. The table displays miRNAs with 10 or more target genes from the microarray data of 1001 miRNAs (S2 Table).

miRNAs	Fold Change	No: of Hits
hsa-miR-125a-5p	-9.69	15
hsa-miR-145-5p	-55.42	14
hsa-miR-151a-3p	-32.1	14
hsa-miR-139-5p	-5.65	13
hsa-miR-24-3p	-3.53	13
hsa-miR-130a-3p	-8.11	13
hsa-miR-155-5p	-3.49	13
hsa-miR-21-5p	-10.11	12
hsa-miR-29a-3p	-8.8	12
hsa-miR-498	2.05	12
hsa-miR-132-3p	-7.55	11
hsa-miR-221-3p	-5.72	11
hsa-miR-130b-3p	-6.11	11
hsa-miR-25-3p	-7.23	11
hsa-miR-337-5p	-4.85	11
hsa-let-7b-5p	-7.73	11
hsa-miR-382-5p	-13.14	10
hsa-miR-199a-5p	-11.9	10
hsa-miR-140-3p	-3.81	10
hsa-miR-532-5p	-4.02	10
hsa-miR-122-5p	-4.69	10
	**Total**	**247**

**Table 3:** Highly altered miRNAs associated with COL1A2, COL3A1, MMP9 and MMP2 genes as determined by miRNA array of Group 1 (n = 4) vs Group 2 (n = 4). The screening was narrowed down to seven by considering the fold-change below -9 and above +3.

miRNAs	Fold Change	No: of Hits
hsa-miR-145-5p	-55.42	14
hsa-miR-151a-3p	-32.1	14
hsa-miR-382-5p	-13.14	10
hsa-miR-199a-5p	-11.9	10
hsa-miR-21-5p	-10.11	12
hsa-miR-125a-5p	-9.69	15
hsa-miR-498	2.05	12
	**Total**	**87**

## Discussion

Tendon tissue is composed of parallel arrays of collagen fibers and elongated tenocytes entangled in the ECM matrix [14]. The repair responses to tendon injuries proceed via a series of overlapping and dynamic physiological events including migration of immune cells, inflammation, and ECM remodeling which will ultimately end up in scarring. ECM breakdown by MMPs is another hallmark for ECM remodeling occurring at the site [15]. Fibrillar collagen (especially collagen type 1 and type 3) degradation is mainly catalyzed by MMP1, MMP8 and MMP13 by the cleavage of peptide bond between Gly775 and Leu776, which disturbs the triple helical structure which enables the collagen chains to unwind. The cleaved fragments denature to form gelatin and are degraded by gelatinases, MMP2 and MMP9 [15]. Thus, the hyperactivity of MMP9 or MMP2 can be considered as a manifestation of the extent of ECM damage in pathological conditions.

Even after the ECM remodeling is complete, tendons may take several months to be functionally active as before. The collagen fibers of smaller diameter were found increased at the site of the injury which results in the failure of load bearing functions [16]. Such small diameter fibers were reported to be of collagen type 3 and are secreted by fibroblasts/tenoblasts after about 7 days of injury. These fibers will be concentrated at the lesion site and form a disorganized network which can be visualized through H&E, NFR and pentachrome staining [14]. Subsequently, these fibers orient along the longitudinal axis of the tendons and the fibroblast activity will be diminished which favors the collagen type 1 deposition to restore the collagen 1 to 3 ratio [17]. But, the actual biological events and molecular mechanisms underlying the switching of collagen type 1 over collagen type 3 largely remain unknown.

MiRNA mediated mechanisms have been reported to have a prominent role in controlling the cellular phenotypes and the composition of the ECM during injury and repair. There are over 30 collagen genes that codes for more than 20 different types of collagen molecules which are under the influence of several miRNAs [18]. Being key constituents of tendon, collagen type 1 and collagen type 3 are the primary focus of this study. Collagen type 1 was reported to be regulated by miR-133a, miR-29b and miR-29c in cancer cells [19]. Similarly, miR-29, miR-218, miR-340 and miR206 were reported to regulate MMP2 and MMP9 and these effects were also profound in cancer cells [18]. Recent reports have shown that miR-29 is a significant mediator for the regulation of collagen type 1 and type 3, and IL-33 in supraspinatus tendon [20]. Interestingly, miR-29 was found to be downregulated (fold change– 8.8) in our arthritis group. There are reports showing that TGF-β signaling significantly downregulates miR-29 in clinical conditions like cardiac fibrosis [21, 22]. Also, TGF-β signaling aggravates osteoarthritis by associating with Smad signaling [23]. The enhanced level of TGF-β in the arthritis environment could be a reason for the downregulation of miR-29 in our arthritis group. Still, the involvement of multiple genes and the associated miRNA regulators has not yet been reported. Our attempt was to screen major miRNAs associated with the genes interconnecting the key regulatory genes of tendon matrix, COL1A2, COL3A1, MMP9 and MMP2. Since the arthritic environment aggravates the disorganization of tendon matrix, we compared arthritis group (Group 1) with non-arthritis group (Group 2) using miRNA array to elucidate the miRNAs associated with shoulder biceps matrix organization. Combining our data with NetworkAnalyst led to identification of seven prominent miRNAs of the tendon matrisome which could have implications in the tendon-related pathologies.

The microRNA hsa-miR-145-5p was found to be downregulated with a -55.42 fold change in Group 1 patients and was found to have 14 target genes associated with tendon matrix organization. This suggests that hsa-miR-145-5p is required for proper maintenance of ECM structure and composition as it was evident from increased disorganization and decreased collagen type 1 to type 3 ratio. The microRNA miR-145-5p was reported to be involved in the maintenance of vessel wall thickness and its absence results in disorientation of ECM and subsequent vessel wall thinning [24]. The upregulation of miR-145-5p was associated with ECM remodeling also. Moreover, miR-145-5p was found to control collagen type 3 expression via TGF-β activation pathway [25]. This could be applicable to our findings as well, because of the down regulation of miR-145-5p during disorganization of the tendon matrix.

The microRNA hsa-miR-151a-3p was found to be downregulated (-32.1 fold change) in Group 1 tendons suggesting its role in ECM remodeling. MMP2, MMP9, TIMP4, HDAC1, RELA, SP1, TP53, and other molecules were found to be the potential targets of hsa-miR-151a-3p and the gene products are the active players of ECM remodeling [26]. The microRNA hsa-miR-151a-3p was involved in cardiac hypertrophy, carcinogenesis and polycystic ovary [26]. But, the reports regarding the role of hsa-miR-151a-3p in human shoulder tendinopathies are limited in the literature. Moreover, upregulation of miR-151a-3p in centenarian population signifies its role in longevity which might have implications with the maintenance of ECM integrity and the composition of the matrisome [27]. Being aggravated with age, the elucidation of miR-151a-3p action, mechanism, and mediators involved in shoulder tendinopathies appears to be advantageous for the development of novel therapies.

The microRNA hsa-miR-382-5p was found to regulate 10 genes associated with tendon matrisome and its major targets include MMP2, FOS, FOSL1. TGF-β1-mediated induction of miR-382-5p in mouse kidneys was found to inhibit kallikrin-5 which is catalyzed by the degradation of ECM proteins [28]. Superoxide dismutase 2 (SOD2) was identified to be one of the direct targets of miR-382 suggesting their protective role in oxidative stress. The inhibition of miR-382 upregulated E-cadherin expression and altered the cellular phenotypes in diabetic kidneys [26, 29]. In cancer, miR-382 was shown to suppress osteosarcoma metastasis by targeting YB-1 oncogene [30, 31]. The regulatory roles of hsa-miR-382-5p in tendon biology remain unexplored and in our study, downregulation of hsa-miR-382-5p and was found to hold a strong correlation with increased ECM damage in Group 1 tendons suggesting its role in maintaining tendon ECM integrity.

The microRNA hsa-miR-199a-5p was predicted to affect the expression of ten genes which have JUN, NF-κβ, MMP2, and CKCL6 as common targets. miR-199a species attained prior attention of cancer researchers owing to their contrast features as it can suppress or progress cancer depending on the tissue type [32]. The cells treated with miR-199a-5p were channeled to apoptotic pathways and also underwent an unusual form of cell death called methuosis, which is characterized by vacuole formation and macropinocytosis [33]. The miR-199 family of microRNAs have also been identified to regulate HIF-1α and p53 to facilitate apoptosis [34]. However, little is known about their functional role in tendon tissues.

The microRNA hsa-miR-21-5p was found to regulate twelve target genes which had MMP2, MMP9, FOS, FOSL1, LGALS3, LGALS8, and SP1 as common targets. The miR-21 is the first miRNA biomarker, discovered in B cell lymphoma patients and the serum levels of miR-21 was correlated with relapse-free survival [35]. The miR-21 family has been significantly examined for tumor metastasis without affecting cell proliferation and targets myristoylated alanine rich protein kinase C substrate (MARCKS), a protein which is associated with actin cytoskeleton. Moreover, miR-21 was also shown to regulate Akt and ERK pathways and activate angiogenesis through HIF-1α [36]. The mRNA encoding PTEN is another direct target of miR-21 and since MMP2 and MMP9 are downstream mediators of PTEN pathway, miR-21 was presumed to indirectly regulate the expression of MMP2 and MMP9 [37]. In addition, the ability of miR-21 to induce angiogenesis was identified to be mediated through upregulation of VEGF [38]. In contrast, miR-21 promotes fibrosis in lung tissue which is mediated through TGF-β1 and the ECM protein fibronectin in lung tissue [39]. Another trigger for miR-21 expression is c-Fos which prevents apoptosis via programmed cell death 4 (PDCD4) that is prevalent in bone tissue where miR-21 mediates particle-induced osteolysis [40]. However, the functional role of miR-21 warrants further investigation.

The microRNA hsa-miR-125a-5p in this study identified to have fifteen targets which include the well-known targets NF-κβ, SP1, and MMP2. The miR-125a-5p has been reported to be downregulated in male breast cancer patients with respect to tumor ErbB2 levels [40, 41]. TRAF6 was identified as one of the direct targets for miR-125a and after activation by NF-κβ, NFATc1 binds to miR-125a promoter to inhibit TRAF6 [40]. Furthermore, hsa-miR-125a-5p downregulates HBV (hepatitis B virus) surface antigen and arrests secretion of HBsAg. Further investigations led to the hypothesis that apart from immune system, hsa-miR-125a-5p also confers defense against HBV infection [42]. The extrapolation of hsa-miR-125a-5p to human tendon tissues can pave way for the elucidation of their functional role in tendinopathies.

The microRNA hsa-miR-498 was found to be upregulated in this study and it had twelve target genes which include HDAC1, HDAC2, COL1A1, and JUN. Since COL1A1 is one of the targets, upregulation of hsa-miR-498 could be one of the reasons for the downregulation of collagen type 1 and alteration in the collagen type 1 to collagen type 3 ratio. Hypoxia contributes to the triggering of the expression of miR-498 in cancer microenvironment [43]. Indeed, hypoxia is a key contributing factor of shoulder injury and tenocytes subjected to hypoxia would undergo apoptosis along with ECM damage [44]. Hypoxia and ischemia in the tendon might have induced miR-498 which downregulated collagen type 1 expression but, additional studies are required to validate this aspect. In addition, miRNA-498 was found to be upregulated to control the metastasis of several cancer types including liver cancer, adenocarcinoma and retinoblastoma [45]. Also, increased expression of miR-498 has been correlated with the ECM damage associated with psoriasis [46].

The specific interactions and functional role of miRNAs in shoulder tendons have not been well established. Epigenetic alterations in gene expression could have significant implications on the initiation and progression of shoulder tendinopathies. Little information is available in the literature regarding the screening of miRNAs with respect to the pathological events associated with the shoulder tendon. ECM disorganization and alterations of collagen type 1 to type 3 ratio have been considered to be a hallmark of tendon injury and the quantification of these collagen types can give the status of repair responses. We carried out miRNA array to screen and identify key miRNAs associated with the ECM remodeling which correlates with our histological results. We examined the long head of the biceps in patients with and without arthritis because in the arthritic environment the ECM damage was found to be aggravated. Most of the key miRNAs were found downregulated in arthritis group (Group 1) indicating their requirement to maintain the integrity of tendon matrisome. Lack of normal control specimen, heterogeneity and variation among the patients, lesser RNA yield, and limited sample availability were the limitations in the study. Our approach of comparing Group 1 and Group 2 samples along with NetworkAnalyst has revealed seven miRNAs which are presumed to play a predominant role in tendon pathology and the understanding of their mechanism of action and functions in both *in vitro* and *in vivo* models can provide new insights for the exploitation of these findings to therapeutic arena.

## Methodology

### RCI specimen preparation

The study was approved by the Creighton University Institutional Review Board. The study protocol and the details of the procedures were explained to the participants in layman terms, and the written informed consent and the HIPPA forms were signed by the volunteers prior to the surgery for collection of tissue.

The long head of the biceps tendon of around 3cm length removed from 8 patients undergoing shoulder surgery and the tendon tissue was collected in UW (*University of Wisconsin*) solution. Four out of eight patients demonstrated severe glenohumeral arthritis (Group 1) and the control group did not have any radiographic or gross evidence of glenohumeral arthritis (Group 2). One portion of the tissue was fixed in formalin and the rest was used for RNA isolation and microarray analysis. The formalin fixed tissues were embedded in paraffin and 5 μm thick sections were cut using a microtome and used for histological staining.

### Histology

The sections were deparaffinized and stained with nuclear fast red (NFR) stain and hematoxylin and eosin (H&E) to determine tissue morphology. The organization and arrangement of collagen fibers and other ECM components were monitored by Movat pentachrome staining. After mounting (with xylene based mounting media for H&E, and pentachrome staining and water based media for NFR staining), the tissue sections were imaged using an inverted microscope attached with an imaging camera (Olympus BX51; Olympus America, Center Valley, PA), [47–49].

### Immunofluorescence

The tissue sections were analyzed for the expression of Collagen I and Collagen III and MMP-2 and MMP-9 expression by immuno-double staining following our previously established standard protocols [13, 45, 50]. HIER buffer (Heat Induced Epitope Retrieval) was used for antigen retrieval at 95°C for 20 mins and the sections blocked in PBS containing 0.25% Triton X-100 and 5% horse serum for 2 hr at room temperature. Primary antibody cocktails used were human anti-mouse collagen I and human anti-rabbit collagen III, human anti-mouse MMP2 and human anti-rabbit MMP9 diluted at 1:50 in PBS. Fluorochrome-conjugated secondary antibodies were either donkey anti-mouse or goat anti-rabbit at a dilution of about 1:200 in PBS. Nuclei were counterstained with 4′,6-diamidino-2-phenylindole (DAPI) and imaged using a fluorescent microscope (Olympus BX51; Olympus America, Center Valley, PA). The fluorescence intensity was quantified using ImageJ software. A negative control with secondary antibodies alone was used to adjust the background and exposure limits.

### RNA isolation from tendon

Tendon tissue from proximal portion of the biceps (weighing around 200mg) was homogenized in TRIZOL solution and RNA was isolated following the manufacturers guidelines. The isolated RNA was suspended in RNase free water and quantified using Nanodrop 2000. The RNA quality was analyzed using bioanalyzer to obtain the RIN score before hybridizing the samples onto the miRNA microarray (miRNA 4.0 array). Due to the nature of the tissue, the RIN score of the isolated RNA was between (2.1 to 4.8). RNA samples were processed for microarray analysis at Kansas University Medical Center and the raw data was analyzed using Expression Console software from Alegent. Microarray analysis was carried out in two separate batches. Two samples from each group were tested in each batch and the data was analyzed. Our analysis showed consistent results in the expression profiles from both the batches indicating reproducibility and pathological specificity in the samples.

### Gene-crosstalk evaluation using NetworkAnalyst

The crosstalk among the genes associated with ECM disorganization from our immunofluorescence data was assessed by using NetworkAnalyst program based on published data base [51, 52]. COL1A2, COL3A1, MMP9 and MMP2 were used as input for NetworkAnalyst and the individual genes that were identified as potential mediators were evaluated individually for their target miRNAs from the miRNA array data.

### Statistical analysis

Each group consisted of four patients. The results of collagen type 1 to type 3 ratios were expressed as mean ± SEM and the statistical significance was evaluated by unpaired t test using one-way ANOVA. The graphs are generated using GraphPad Prism 7 software. The differences in the expression levels were considered statistically significant if the p-values were less than 0.05 (*p<0*.*05*).

### Conclusions

ECM disorganization is one of the hallmarks of tendinopathy which is under the influence of epigenetic regulation by miRNAs. The key miRNAs associated with biceps tendon within an arthritic environment were identified using miRNA microarray and the results were compared with that of patients with and without glenohumeral arthritis. With COL1A2, COL3A1, MMP9 and MMP2 selected as the main regulatory genes, NetworkAnalyst data revealed 81 gene products and 100 pathways associated with shoulder tendon matrisome disorganization. Seven miRNAs were found to be highly active and were thought to mediate the major biochemical events associated with the integrity of tendon matrisome. The elucidation of their mechanism of action and functions in both *in vitro* and *in vivo* can improve the knowledge of tendon pathology which can open novel therapeutic opportunities for tendon specific disorders.

## Supporting Information

S1 Table Pathways associated with COL1A2, COL3A1, MMP9 and MMP2 as determined by NetworkAnalyst. (DOCX)

S2 Table 1001 miRNAs associated with the regulation of 81 genes connected with COL1A2, COL3A1, MMP9 and MMP2. (DOCX)
