# Supplementary material for: MicroRNAs Associated with Shoulder Tendon Matrisome Disorganization in Glenohumeral Arthritis
Source: PLoS One. 2016 Dec 16;11(12):e0168077. doi: 10.1371/journal.pone.0168077 (PMC5161352; doi:10.1371/journal.pone.0168077)
Supplement: S1 Table — (DOCX) [file pone.0168077.s001.docx]

| 1. Extracellular matrix organization |
| --- |
| 1. Hemostasis |
| 1. Signal Transduction |
| 1. Integrin cell surface interactions |
| 1. Platelet Adhesion to exposed collagen |
| 1. Degradation of the extracellular matrix |
| 1. Chemokine receptors bind chemokines |
| 1. Degradation of collagen |
| 1. Activation of Gene Expression by SREBP (SREBF) |
| 1. Activation of the AP-1 family of transcription factors |
| 1. Assembly of collagen fibrils and other multimeric structures |
| 1. Activation of Matrix Metalloproteinases |
| 1. Cell surface interactions at the vascular wall |
| 1. TRAF6 Mediated Induction of proinflammatory cytokines |
| 1. Fibronectin matrix formation |
| 1. Signaling by TGF-beta Receptor Complex |
| 1. Anchoring fibril formation |
| 1. Regulation of Cholesterol Biosynthesis by SREBP (SREBF) |
| 1. Toll Like Receptor 10 (TLR10) Cascade |
| 1. Toll Like Receptor 5 (TLR5) Cascade |
| 1. MyD88 cascade initiated on plasma membrane |
| 1. TRAF6 mediated induction of NFkB and MAP kinases upon TLR7/8 or 9 activation |
| 1. Regulation of Lipid Metabolism by Peroxisome proliferator-activated receptor alpha (PPARalpha) |
| 1. Toll Like Receptor 7/8 (TLR7/8) Cascade |
| 1. MyD88 dependent cascade initiated on endosome |
| 1. Transcriptional activity of SMAD2/SMAD3:SMAD4 heterotrimer |
| 1. Toll Like Receptor 9 (TLR9) Cascade |
| 1. MyD88:Mal cascade initiated on plasma membrane |
| 1. Toll Like Receptor TLR1:TLR2 Cascade |
| 1. Toll Like Receptor TLR6:TLR2 Cascade |
| 1. Toll Like Receptor 2 (TLR2) Cascade |
| 1. Elastic fiber formation |
| 1. p75 NTR receptor-mediated signaling |
| 1. Collagen formation |
| 1. TRIF-mediated TLR3/TLR4 signaling |
| 1. MyD88-independent cascade |
| 1. Toll Like Receptor 3 (TLR3) Cascade |
| 1. NOTCH1 Intracellular Domain Regulates Transcription |
| 1. Platelet degranulation |
| 1. Constitutive Signaling by NOTCH1 HD+PEST Domain Mutants |
| 1. Downregulation of SMAD2/3:SMAD4 transcriptional activity |
| 1. Response to elevated platelet cytosolic Ca2+ |
| 1. p75NTR negatively regulates cell cycle via SC1 |
| 1. Activated TLR4 signalling |
| 1. Constitutive Signaling by NOTCH1 PEST Domain Mutants |
| 1. PPARA Activates Gene Expression |
| 1. Developmental Biology |
| 1. Toll Like Receptor 4 (TLR4) Cascade |
| 1. Factors involved in megakaryocyte development and platelet production |
| 1. SMAD2/SMAD3:SMAD4 heterotrimer regulates transcription |
| 1. Platelet activation, signaling and aggregation |
| 1. TGF-beta receptor signaling activates SMADs |
| 1. MAPK targets/ Nuclear events mediated by MAP kinases |
| 1. CHL1 interactions |
| 1. RIG-I/MDA5 mediated induction of IFN-alpha/beta pathways |
| 1. Regulation of Gene Expression by Hypoxia-inducible Factor |
| 1. Signal transduction by L1 |
| 1. Toll-Like Receptors Cascades |
| 1. Signaling by NOTCH1 t(7;9)(NOTCH1:M1580_K2555) Translocation Mutant |
| 1. Signaling by NOTCH1 in Cancer |
| 1. Signaling by NOTCH1 PEST Domain Mutants in Cancer |
| 1. FBXW7 Mutants and NOTCH1 in Cancer |
| 1. Signaling by NOTCH1 HD Domain Mutants in Cancer |
| 1. Signaling by NOTCH1 HD+PEST Domain Mutants in Cancer |
| 1. Signaling by NOTCH1 |
| 1. Regulated proteolysis of p75NTR |
| 1. Molecules associated with elastic fibres |
| 1. Crosslinking of collagen fibrils |
| 1. RIP-mediated NFkB activation via DAI |
| 1. Peptide ligand-binding receptors |
| 1. Signaling by Activin |
| 1. Fatty acid, triacylglycerol, and ketone body metabolism |
| 1. DAI mediated induction of type I IFNs |
| 1. NF-kB is activated and signals survival |
| 1. TRAF3-dependent IRF activation pathway |
| 1. Signaling by NOTCH |
| 1. p75NTR signals via NF-kB |
| 1. TRAF6 mediated NF-kB activation |
| 1. Signaling by NODAL |
| 1. MAP kinase activation in TLR cascade |
| 1. Cytosolic sensors of pathogen-associated DNA |
| 1. L1CAM interactions |
| 1. Collagen biosynthesis and modifying enzymes |
| 1. TAK1 activates NFkB by phosphorylation and activation of IKKs complex |
| 1. G alpha (i) signalling events |
| 1. Interferon alpha/beta signaling |
| 1. Influenza Virus Induced Apoptosis |
| 1. Innate Immune System |
| 1. Cellular responses to stress |
| 1. Cellular response to hypoxia |
| 1. Downregulation of TGF-beta receptor signaling |
| 1. Regulation of Hypoxia-inducible Factor (HIF) by Oxygen |
| 1. TRAF6 mediated IRF7 activation |
| 1. Localization of the PINCH-ILK-PARVIN complex to focal adhesions |
| 1. Axon guidance |
| 1. Basigin interactions |
| 1. GPVI-mediated activation cascade |
| 1. Class A/1 (Rhodopsin-like receptors) |
| 1. Host Interactions with Influenza Factors |
| 1. Immune System |

**S1 Table**: Pathways associated with COL1A2, COL3A1, MMP9 and MMP2 as determined by NetworkAnalyst.
