# Supplementary material for: MicroRNAs Associated with Shoulder Tendon Matrisome Disorganization in Glenohumeral Arthritis
Source: PLoS One. 2016 Dec 16;11(12):e0168077. doi: 10.1371/journal.pone.0168077 (PMC5161352; doi:10.1371/journal.pone.0168077)
Supplement: S2 Table — (DOCX) [file pone.0168077.s002.docx]

| **miRNAs** | **F.C** | **miRNAs** | **F.C** | **miRNAs** | **F.C** |
| --- | --- | --- | --- | --- | --- |
| **MMP2** |  | **MMP9** |  | **THBS1** |  |
| hsa-miR-361-5p | -69.26 | hsa-miR-151a-3p | -32.1 | hsa-miR-1273g-3p | -64.17 |
| hsa-miR-145-5p | -55.42 | hsa-let-7e-5p | -11.7 | hsa-miR-151a-3p | -32.1 |
| hsa-miR-23b-3p | -35.32 | hsa-miR-451a | -10.45 | hsa-miR-132-3p | -7.55 |
| hsa-miR-146a-5p | -29.52 | hsa-miR-21-5p | -10.11 | hsa-miR-30a-3p | -5.01 |
| hsa-miR-193b-3p | -20.35 | hsa-miR-339-5p | -7.22 | hsa-miR-377-5p | -3.56 |
| hsa-miR-30d-5p | -19.32 | hsa-miR-491-5p | -6.22 | hsa-miR-297 | 3.81 |
| hsa-miR-486-5p | -15.04 | hsa-miR-652-3p | -5.88 | **SP3** |  |
| hsa-miR-422a | -14.1 | hsa-miR-494-3p | -4.06 | hsa-miR-191-5p | -71.26 |
| hsa-miR-26a-5p | -13.97 | hsa-miR-345-5p | -3.98 | hsa-miR-361-5p | -69.26 |
| hsa-miR-382-5p | -13.14 | hsa-miR-24-3p | -3.53 | hsa-miR-99b-5p | -59.57 |
| hsa-miR-199a-5p | -11.9 | hsa-miR-92b-5p | -3.26 | hsa-miR-145-5p | -55.42 |
| hsa-miR-30a-5p | -11.48 | **COL1A2** |  | hsa-miR-99a-5p | -51.68 |
| hsa-miR-451a | -10.45 | hsa-miR-26a-5p | -13.97 | hsa-miR-100-5p | -51.6 |
| hsa-miR-151a-5p | -10.22 | hsa-miR-29a-3p | -8.8 | hsa-miR-151a-3p | -32.1 |
| hsa-miR-21-5p | -10.11 | hsa-miR-25-3p | -7.23 | hsa-let-7a-5p | -31.63 |
| hsa-miR-125a-5p | -9.69 | hsa-miR-337-5p | -4.85 | hsa-miR-22-3p | -30.84 |
| hsa-miR-29a-3p | -8.8 | hsa-miR-1343-5p | -3.92 | hsa-miR-409-3p | -28.06 |
| hsa-miR-106b-5p | -8.08 | hsa-miR-6870-5p | 3.95 | hsa-miR-127-3p | -26.62 |
| hsa-miR-30c-5p | -7.67 | **COL13A1** |  | hsa-miR-30d-5p | -19.32 |
| hsa-miR-132-3p | -7.55 | hsa-miR-29a-3p | -8.8 | hsa-miR-486-5p | -15.04 |
| hsa-miR-663a | -7.43 | hsa-let-7b-5p | -7.73 | hsa-miR-103a-3p | -14.87 |
| hsa-miR-339-5p | -7.22 | hsa-miR-143-3p | -4.17 | hsa-miR-134-5p | -14.82 |
| hsa-miR-193a-3p | -7.13 | hsa-miR-455-3p | -3.45 | hsa-miR-23a-3p | -14.18 |
| hsa-miR-193b-5p | -6.85 | **SP1** |  | hsa-miR-382-5p | -13.14 |
| hsa-miR-708-5p | -6.41 | hsa-miR-361-5p | -69.26 | hsa-miR-10b-5p | -12.27 |
| hsa-miR-491-5p | -6.22 | hsa-miR-99b-5p | -59.57 | hsa-miR-107 | -11.99 |
| hsa-miR-221-3p | -5.72 | hsa-miR-145-5p | -55.42 | hsa-miR-30a-5p | -11.48 |
| hsa-miR-139-5p | -5.65 | hsa-miR-99a-5p | -51.68 | hsa-miR-193a-5p | -10.93 |
| hsa-miR-4443 | -5.45 | hsa-miR-100-5p | -51.6 | hsa-miR-574-3p | -10.54 |
| hsa-miR-324-3p | -5.2 | hsa-miR-23b-3p | -35.32 | hsa-miR-451a | -10.45 |
| hsa-miR-30a-3p | -5.01 | hsa-miR-151a-3p | -32.1 | hsa-miR-151a-5p | -10.22 |
| hsa-miR-20a-5p | -4.67 | hsa-let-7a-5p | -31.63 | hsa-miR-125a-5p | -9.69 |
| hsa-miR-197-3p | -4.03 | hsa-miR-22-3p | -30.84 | hsa-miR-370-3p | -8.38 |
| hsa-miR-15b-5p | -3.98 | hsa-let-7d-5p | -29.9 | hsa-miR-28-5p | -8.31 |
| hsa-miR-140-3p | -3.81 | hsa-miR-146a-5p | -29.52 | hsa-miR-30c-5p | -7.67 |
| hsa-miR-10b-3p | -3.7 | hsa-miR-409-3p | -28.06 | hsa-miR-193a-3p | -7.13 |
| hsa-miR-24-3p | -3.53 | hsa-miR-127-3p | -26.62 | hsa-miR-27b-5p | -6.64 |
| hsa-miR-92b-3p | -3.43 | hsa-miR-150-5p | -23.41 | hsa-miR-708-5p | -6.41 |
| hsa-miR-452-5p | -3.06 | hsa-miR-181a-5p | -21.34 | hsa-miR-221-3p | -5.72 |
| **COL1A1** |  | hsa-miR-193b-3p | -20.35 | hsa-miR-381-3p | -5.67 |
| hsa-miR-193a-5p | -10.93 | hsa-miR-30d-5p | -19.32 | hsa-miR-139-5p | -5.65 |
| hsa-miR-532-3p | -7.12 | hsa-miR-342-3p | -18.02 | hsa-miR-874-3p | -5.31 |
| hsa-miR-193b-5p | -6.85 | hsa-miR-199a-3p | -17.88 | hsa-miR-362-5p | -5.1 |
| hsa-miR-491-5p | -6.22 | hsa-miR-486-5p | -15.04 | hsa-miR-665 | -5.1 |
| hsa-miR-143-3p | -4.17 | hsa-miR-103a-3p | -14.87 | hsa-miR-487b-3p | -5.06 |
| hsa-miR-498 | 2.05 | hsa-miR-134-5p | -14.82 | hsa-miR-337-5p | -4.85 |
| **IRF1** |  | hsa-miR-31-5p | -14.44 | hsa-miR-122-5p | -4.69 |
| hsa-miR-193b-3p | -20.35 | hsa-miR-23a-3p | -14.18 | hsa-miR-17-3p | -4.6 |
| hsa-miR-23a-3p | -14.18 | hsa-miR-422a | -14.1 | hsa-miR-342-5p | -4.46 |
| hsa-miR-28-5p | -8.31 | hsa-miR-195-5p | -14.04 | hsa-miR-671-5p | -4.45 |
| hsa-miR-130a-3p | -8.11 | hsa-miR-26a-5p | -13.97 | hsa-miR-10a-5p | -4.02 |
| hsa-miR-132-3p | -7.55 | hsa-miR-382-5p | -13.14 | hsa-miR-532-5p | -4.02 |
| hsa-miR-193a-3p | -7.13 | hsa-let-7c-5p | -12.58 | hsa-miR-345-5p | -3.98 |
| hsa-miR-130b-3p | -6.11 | hsa-miR-497-5p | -12.3 | hsa-miR-143-5p | -3.9 |
| hsa-miR-24-2-5p | -5.92 | hsa-miR-10b-5p | -12.27 | hsa-miR-140-3p | -3.81 |
| hsa-miR-93-5p | -4.15 | hsa-miR-107 | -11.99 | hsa-miR-10b-3p | -3.7 |
| hsa-miR-345-5p | -3.98 | hsa-miR-199a-5p | -11.9 | hsa-miR-127-5p | -3.33 |
| hsa-miR-143-5p | -3.9 | hsa-let-7e-5p | -11.7 | hsa-miR-654-3p | -3.23 |
| hsa-miR-140-3p | -3.81 | hsa-miR-30a-5p | -11.48 | hsa-miR-452-5p | -3.06 |
| hsa-miR-24-3p | -3.53 | hsa-miR-193a-5p | -10.93 | hsa-miR-498 | 2.05 |
| hsa-miR-92b-3p | -3.43 | hsa-miR-15a-5p | -10.66 | hsa-miR-6831-5p | 2.27 |
| hsa-miR-1307-3p | -3.2 | hsa-miR-574-3p | -10.54 | hsa-miR-4487 | 2.3 |
| hsa-miR-378a-5p | -3 | hsa-miR-451a | -10.45 | hsa-miR-6124 | 3.85 |
| hsa-miR-7107-5p | 2.87 | hsa-miR-500a-3p | -10.25 | **CAND1** |  |
| **RELA** |  | hsa-miR-151a-5p | -10.22 | hsa-miR-191-5p | -71.26 |
| hsa-miR-151a-3p | -32.1 | hsa-miR-21-5p | -10.11 | hsa-miR-99b-5p | -59.57 |
| hsa-miR-22-3p | -30.84 | hsa-miR-125a-5p | -9.69 | hsa-miR-99a-5p | -51.68 |
| hsa-miR-339-3p | -5.8 | hsa-miR-29a-3p | -8.8 | hsa-miR-100-5p | -51.6 |
| hsa-miR-324-5p | -4.31 | hsa-let-7i-5p | -8.38 | hsa-miR-425-5p | -33.79 |
| **EGR1** |  | hsa-miR-370-3p | -8.38 | hsa-miR-30d-5p | -19.32 |
| hsa-miR-191-5p | -71.26 | hsa-miR-28-5p | -8.31 | hsa-miR-195-5p | -14.04 |
| hsa-miR-150-5p | -23.41 | hsa-miR-130a-3p | -8.11 | hsa-miR-30a-5p | -11.48 |
| hsa-miR-195-5p | -14.04 | hsa-miR-214-5p | -8.1 | hsa-miR-29a-3p | -8.8 |
| hsa-miR-497-5p | -12.3 | hsa-miR-106b-5p | -8.08 | hsa-miR-30c-5p | -7.67 |
| hsa-miR-15a-5p | -10.66 | hsa-miR-16-5p | -7.98 | hsa-miR-339-5p | -7.22 |
| hsa-miR-21-5p | -10.11 | hsa-let-7b-5p | -7.73 | hsa-miR-339-3p | -5.8 |
| hsa-miR-125a-5p | -9.69 | hsa-miR-30c-5p | -7.67 | hsa-miR-139-5p | -5.65 |
| hsa-miR-25-3p | -7.23 | hsa-miR-132-3p | -7.55 | hsa-miR-99b-3p | -5.02 |
| hsa-miR-339-5p | -7.22 | hsa-miR-663a | -7.43 | **LCN2** |  |
| hsa-miR-4443 | -5.45 | hsa-miR-25-3p | -7.23 | hsa-miR-29a-3p | -8.8 |
| hsa-miR-1233-5p | -5.09 | hsa-miR-339-5p | -7.22 | hsa-miR-370-3p | -8.38 |
| hsa-miR-7977 | -4.67 | hsa-miR-146b-5p | -7.14 | hsa-miR-491-5p | -6.22 |
| hsa-miR-15b-5p | -3.98 | hsa-miR-532-3p | -7.12 | hsa-miR-139-5p | -5.65 |
| hsa-miR-24-3p | -3.53 | hsa-miR-744-5p | -6.94 | hsa-miR-324-5p | -4.31 |
| hsa-miR-92b-3p | -3.43 | hsa-miR-193b-5p | -6.85 | hsa-miR-106b-3p | -3.33 |
| hsa-miR-4646-5p | -3.25 | hsa-miR-708-5p | -6.41 | **TGFB1** |  |
| hsa-miR-619-5p | -3.16 | hsa-miR-491-5p | -6.22 | hsa-miR-361-5p | -69.26 |
| hsa-miR-4668-5p | 3.73 | hsa-miR-130b-3p | -6.11 | hsa-miR-199a-3p | -17.88 |
| **MYOC** |  | hsa-miR-24-2-5p | -5.92 | hsa-miR-134-5p | -14.82 |
| hsa-miR-151a-3p | -32.1 | hsa-miR-652-3p | -5.88 | hsa-miR-28-5p | -8.31 |
| hsa-miR-486-5p | -15.04 | hsa-miR-339-3p | -5.8 | hsa-miR-663a | -7.43 |
| hsa-miR-665 | -5.1 | hsa-miR-381-3p | -5.67 | hsa-miR-744-5p | -6.94 |
| hsa-miR-337-5p | -4.85 | hsa-miR-139-5p | -5.65 | hsa-miR-193b-5p | -6.85 |
| hsa-miR-532-5p | -4.02 | hsa-miR-874-3p | -5.31 | hsa-miR-139-5p | -5.65 |
| hsa-miR-155-5p | -3.49 | hsa-miR-21-3p | -5.23 | **FOS** |  |
| hsa-miR-6124 | 3.85 | hsa-miR-324-3p | -5.2 | hsa-miR-99a-5p | -51.68 |
| **LGALS3** |  | hsa-miR-6722-3p | -5.15 | hsa-miR-181a-5p | -21.34 |
| hsa-miR-151a-3p | -32.1 | hsa-miR-154-5p | -5.1 | hsa-miR-342-3p | -18.02 |
| hsa-miR-151a-5p | -10.22 | hsa-miR-362-5p | -5.1 | hsa-miR-382-5p | -13.14 |
| hsa-miR-21-5p | -10.11 | hsa-miR-665 | -5.1 | hsa-miR-574-3p | -10.54 |
| hsa-miR-30c-5p | -7.67 | hsa-miR-487b-3p | -5.06 | hsa-miR-21-5p | -10.11 |
| hsa-miR-744-5p | -6.94 | hsa-miR-99b-3p | -5.02 | hsa-miR-29a-3p | -8.8 |
| hsa-miR-324-3p | -5.2 | hsa-miR-30a-3p | -5.01 | hsa-miR-130a-3p | -8.11 |
| hsa-miR-30a-3p | -5.01 | hsa-miR-28-3p | -4.94 | hsa-miR-214-5p | -8.1 |
| hsa-miR-27a-3p | -4.89 | hsa-miR-337-5p | -4.85 | hsa-let-7b-5p | -7.73 |
| hsa-miR-17-3p | -4.6 | hsa-miR-6779-5p | -4.78 | hsa-miR-663a | -7.43 |
| hsa-miR-671-5p | -4.45 | hsa-miR-122-5p | -4.69 | hsa-miR-652-3p | -5.88 |
| hsa-miR-151b | -4.2 | hsa-miR-20a-5p | -4.67 | hsa-miR-221-3p | -5.72 |
| hsa-miR-197-3p | -4.03 | hsa-miR-7977 | -4.67 | hsa-miR-139-5p | -5.65 |
| hsa-miR-432-5p | -4.02 | hsa-miR-17-3p | -4.6 | hsa-miR-665 | -5.1 |
| hsa-miR-1343-5p | -3.92 | hsa-miR-342-5p | -4.46 | hsa-miR-6779-5p | -4.78 |
| hsa-miR-127-5p | -3.33 | hsa-miR-671-5p | -4.45 | hsa-miR-20a-5p | -4.67 |
| hsa-miR-493-3p | -3.27 | hsa-miR-324-5p | -4.31 | hsa-miR-7977 | -4.67 |
| **SCUBE3** |  | hsa-miR-2110 | -4.28 | hsa-miR-24-3p | -3.53 |
| hsa-miR-214-5p | -8.1 | hsa-miR-93-5p | -4.15 | hsa-miR-155-5p | -3.49 |
| hsa-miR-6779-5p | -4.78 | hsa-miR-6816-5p | -4.13 | hsa-miR-619-5p | -3.16 |
| hsa-miR-7977 | -4.67 | hsa-miR-494-3p | -4.06 | hsa-miR-139-3p | -3.09 |
| hsa-miR-3197 | -3.89 | hsa-miR-197-3p | -4.03 | hsa-miR-181b-5p | -3.09 |
| hsa-miR-6765-5p | -3.29 | hsa-miR-10a-5p | -4.02 | hsa-miR-937-5p | -3.03 |
| **TIMP2** |  | hsa-miR-532-5p | -4.02 | hsa-miR-6769b-5p | 2.29 |
| hsa-miR-483-5p | 2.56 | hsa-miR-15b-5p | -3.98 | hsa-miR-7107-5p | 2.87 |
| **LAMC2** |  | hsa-miR-345-5p | -3.98 | hsa-miR-8075 | 3.16 |
| hsa-miR-23b-3p | -35.32 | hsa-miR-409-5p | -3.93 | hsa-miR-6870-5p | 3.95 |
| hsa-miR-146a-5p | -29.52 | hsa-miR-143-5p | -3.9 | **CCL7** |  |
| hsa-miR-23a-3p | -14.18 | hsa-miR-664a-5p | -3.89 | hsa-miR-23b-3p | -35.32 |
| hsa-miR-125a-5p | -9.69 | hsa-miR-3197 | -3.89 | hsa-let-7a-5p | -31.63 |
| hsa-miR-29a-3p | -8.8 | hsa-miR-379-5p | -3.88 | hsa-miR-181a-5p | -21.34 |
| hsa-miR-132-3p | -7.55 | hsa-miR-140-3p | -3.81 | hsa-miR-23a-3p | -14.18 |
| **IRF2** |  | hsa-miR-20b-5p | -3.72 | hsa-let-7c-5p | -12.58 |
| hsa-miR-30d-5p | -19.32 | hsa-miR-10b-3p | -3.7 | hsa-let-7e-5p | -11.7 |
| hsa-miR-342-3p | -18.02 | hsa-miR-1909-3p | -3.61 | hsa-let-7b-5p | -7.73 |
| hsa-miR-199a-3p | -17.88 | hsa-miR-377-5p | -3.56 | hsa-miR-381-3p | -5.67 |
| hsa-miR-103a-3p | -14.87 | hsa-miR-17-5p | -3.53 | hsa-miR-99b-3p | -5.02 |
| hsa-miR-31-5p | -14.44 | hsa-miR-24-3p | -3.53 | hsa-miR-324-5p | -4.31 |
| hsa-miR-26a-5p | -13.97 | hsa-miR-155-5p | -3.49 | hsa-miR-143-3p | -4.17 |
| hsa-let-7c-5p | -12.58 | hsa-miR-455-3p | -3.45 | **TFAP2A** |  |
| hsa-miR-10b-5p | -12.27 | hsa-miR-92b-3p | -3.43 | hsa-miR-409-3p | -28.06 |
| hsa-miR-107 | -11.99 | hsa-miR-185-5p | -3.39 | hsa-miR-422a | -14.1 |
| hsa-let-7b-5p | -7.73 | hsa-miR-224-3p | -3.38 | hsa-miR-382-5p | -13.14 |
| hsa-miR-532-3p | -7.12 | hsa-miR-106b-3p | -3.33 | hsa-miR-125a-5p | -9.69 |
| hsa-miR-744-5p | -6.94 | hsa-miR-127-5p | -3.33 | hsa-miR-491-5p | -6.22 |
| hsa-miR-221-3p | -5.72 | hsa-miR-493-3p | -3.27 | hsa-miR-154-5p | -5.1 |
| hsa-miR-381-3p | -5.67 | hsa-miR-92b-5p | -3.26 | hsa-miR-664b-5p | -4.82 |
| hsa-miR-20a-5p | -4.67 | hsa-miR-654-3p | -3.23 | hsa-miR-432-5p | -4.02 |
| hsa-miR-342-5p | -4.46 | hsa-miR-1307-3p | -3.2 | hsa-miR-143-5p | -3.9 |
| hsa-miR-4507 | -4.42 | hsa-miR-106a-5p | -3.18 | hsa-miR-433-3p | -3.89 |
| hsa-miR-10a-5p | -4.02 | hsa-miR-503-5p | -3.17 | hsa-miR-377-5p | -3.56 |
| hsa-miR-432-5p | -4.02 | hsa-miR-6132 | -3.17 | hsa-miR-503-5p | -3.17 |
| hsa-miR-409-5p | -3.93 | hsa-miR-619-5p | -3.16 | hsa-miR-139-3p | -3.09 |
| hsa-miR-155-5p | -3.49 | hsa-miR-181a-3p | -3.14 | hsa-miR-8075 | 3.16 |
| hsa-miR-629-5p | -3.12 | hsa-miR-139-3p | -3.09 | **NFYB** |  |
| hsa-miR-498 | 2.05 | hsa-miR-181b-5p | -3.09 | hsa-miR-181a-5p | -21.34 |
| **ATF2** |  | hsa-miR-452-5p | -3.06 | hsa-miR-30c-5p | -7.67 |
| hsa-miR-199a-3p | -17.88 | hsa-miR-23b-5p | -3.04 | hsa-miR-25-3p | -7.23 |
| hsa-miR-15a-5p | -10.66 | hsa-miR-378a-5p | -3 | hsa-miR-28-3p | -4.94 |
| hsa-miR-451a | -10.45 | hsa-miR-6126 | -3 | hsa-miR-143-3p | -4.17 |
| hsa-miR-21-5p | -10.11 | hsa-miR-8072 | 2.04 | hsa-miR-92b-3p | -3.43 |
| hsa-miR-125a-5p | -9.69 | hsa-miR-498 | 2.05 | **NFYA** |  |
| hsa-miR-29a-3p | -8.8 | hsa-miR-7110-5p | 2.13 | hsa-miR-22-3p | -30.84 |
| hsa-miR-221-3p | -5.72 | hsa-miR-6769b-5p | 2.29 | hsa-miR-150-5p | -23.41 |
| hsa-miR-21-3p | -5.23 | hsa-miR-4487 | 2.3 | hsa-miR-532-3p | -7.12 |
| hsa-miR-30a-3p | -5.01 | hsa-miR-483-5p | 2.56 | hsa-miR-221-3p | -5.72 |
| hsa-miR-122-5p | -4.69 | hsa-miR-7107-5p | 2.87 | hsa-miR-4507 | -4.42 |
| hsa-miR-143-3p | -4.17 | hsa-miR-4668-5p | 3.73 | hsa-miR-140-3p | -3.81 |
| hsa-miR-140-3p | -3.81 | hsa-miR-297 | 3.81 | hsa-miR-629-5p | -3.12 |
| **FOSL1** |  | hsa-miR-6124 | 3.85 | hsa-miR-4668-5p | 3.73 |
| hsa-miR-382-5p | -13.14 | hsa-miR-4467 | 4.09 | **NFYC** |  |
| hsa-miR-21-5p | -10.11 | **JUN** |  | hsa-miR-181a-5p | -21.34 |
| hsa-miR-130a-3p | -8.11 | hsa-miR-342-3p | -18.02 | hsa-miR-199a-5p | -11.9 |
| hsa-let-7b-5p | -7.73 | hsa-miR-199a-3p | -17.88 | hsa-miR-193a-5p | -10.93 |
| hsa-miR-652-3p | -5.88 | hsa-miR-10b-5p | -12.27 | hsa-miR-574-3p | -10.54 |
| hsa-miR-122-5p | -4.69 | hsa-miR-199a-5p | -11.9 | hsa-miR-532-3p | -7.12 |
| hsa-miR-6870-5p | 3.95 | hsa-miR-30a-5p | -11.48 | hsa-miR-221-3p | -5.72 |
| **ESR1** |  | hsa-miR-3178 | -10.86 | hsa-miR-122-5p | -4.69 |
| hsa-miR-145-5p | -55.42 | hsa-miR-15a-5p | -10.66 | hsa-miR-532-5p | -4.02 |
| hsa-miR-99a-5p | -51.68 | hsa-miR-125a-5p | -9.69 | hsa-miR-155-5p | -3.49 |
| hsa-miR-100-5p | -51.6 | hsa-miR-16-5p | -7.98 | hsa-miR-181b-5p | -3.09 |
| hsa-miR-22-3p | -30.84 | hsa-miR-663a | -7.43 | **RFX5** |  |
| hsa-miR-181a-5p | -21.34 | hsa-miR-744-5p | -6.94 | hsa-miR-151a-3p | -32.1 |
| hsa-miR-193b-3p | -20.35 | hsa-miR-139-5p | -5.65 | hsa-let-7d-5p | -29.9 |
| hsa-miR-26a-5p | -13.97 | hsa-miR-4707-5p | -4.31 | hsa-miR-30d-5p | -19.32 |
| hsa-miR-130a-3p | -8.11 | hsa-miR-93-5p | -4.15 | hsa-miR-31-5p | -14.44 |
| hsa-miR-130b-3p | -6.11 | hsa-miR-494-3p | -4.06 | hsa-miR-193a-5p | -10.93 |
| hsa-miR-221-3p | -5.72 | hsa-miR-10a-5p | -4.02 | hsa-let-7i-5p | -8.38 |
| hsa-miR-874-3p | -5.31 | hsa-miR-155-5p | -3.49 | hsa-miR-130a-3p | -8.11 |
| hsa-miR-20b-5p | -3.72 | hsa-miR-139-3p | -3.09 | hsa-miR-30c-5p | -7.67 |
| hsa-miR-181b-5p | -3.09 | hsa-miR-23b-5p | -3.04 | hsa-miR-130b-3p | -6.11 |
| **FLI1** |  | hsa-miR-498 | 2.05 | hsa-miR-409-5p | -3.93 |
| hsa-miR-145-5p | -55.42 | hsa-miR-1908-5p | 3.15 | hsa-miR-379-5p | -3.88 |
| hsa-miR-193b-3p | -20.35 | hsa-miR-297 | 3.81 | **HDAC2** |  |
| hsa-miR-193a-3p | -7.13 | **LGALS8** |  | hsa-miR-145-5p | -55.42 |
| hsa-miR-155-5p | -3.49 | hsa-miR-22-3p | -30.84 | hsa-miR-193a-5p | -10.93 |
| **RFXANK** |  | hsa-miR-127-3p | -26.62 | hsa-miR-130a-3p | -8.11 |
| hsa-miR-145-5p | -55.42 | hsa-miR-21-5p | -10.11 | hsa-miR-132-3p | -7.55 |
| hsa-miR-193a-5p | -10.93 | hsa-miR-139-5p | -5.65 | hsa-miR-130b-3p | -6.11 |
| hsa-miR-130a-3p | -8.11 | hsa-miR-154-5p | -5.1 | hsa-miR-143-3p | -4.17 |
| hsa-miR-132-3p | -7.55 | hsa-miR-337-5p | -4.85 | hsa-miR-455-3p | -3.45 |
| hsa-miR-130b-3p | -6.11 | hsa-miR-409-5p | -3.93 | hsa-miR-127-5p | -3.33 |
| hsa-miR-127-5p | -3.33 | hsa-miR-127-5p | -3.33 | hsa-miR-498 | 2.05 |
| hsa-miR-498 | 2.05 | hsa-miR-92b-5p | -3.26 | **BCAN** |  |
| **PXN** |  | hsa-miR-378a-5p | -3 | hsa-miR-31-5p | -14.44 |
| hsa-miR-199a-3p | -17.88 | **SFTPD** |  | hsa-miR-422a | -14.1 |
| hsa-miR-382-5p | -13.14 | hsa-miR-103a-3p | -14.87 | hsa-miR-199a-5p | -11.9 |
| hsa-miR-199a-5p | -11.9 | hsa-miR-107 | -11.99 | hsa-miR-125a-5p | -9.69 |
| hsa-let-7e-5p | -11.7 | hsa-miR-193a-3p | -7.13 | hsa-miR-132-3p | -7.55 |
| hsa-miR-125a-5p | -9.69 | hsa-miR-324-3p | -5.2 | hsa-miR-744-5p | -6.94 |
| hsa-miR-125b-2-3p | -8.66 | **HDAC1** |  | hsa-miR-487b-3p | -5.06 |
| hsa-miR-221-3p | -5.72 | hsa-miR-191-5p | -71.26 | hsa-miR-99b-3p | -5.02 |
| hsa-miR-433-3p | -3.89 | hsa-miR-99b-5p | -59.57 | hsa-miR-671-5p | -4.45 |
| **ZNF16** |  | hsa-miR-145-5p | -55.42 | hsa-miR-432-5p | -4.02 |
| hsa-miR-361-5p | -69.26 | hsa-miR-99a-5p | -51.68 | hsa-miR-92b-5p | -3.26 |
| hsa-miR-99b-5p | -59.57 | hsa-miR-100-5p | -51.6 | hsa-miR-6124 | 3.85 |
| hsa-miR-145-5p | -55.42 | hsa-miR-151a-3p | -32.1 | **CAPN3** |  |
| hsa-miR-100-5p | -51.6 | hsa-miR-22-3p | -30.84 | hsa-miR-23b-3p | -35.32 |
| hsa-miR-151a-3p | -32.1 | hsa-miR-486-5p | -15.04 | hsa-miR-23a-3p | -14.18 |
| hsa-miR-146a-5p | -29.52 | hsa-miR-23a-3p | -14.18 | hsa-miR-193a-3p | -7.13 |
| hsa-miR-134-5p | -14.82 | hsa-miR-422a | -14.1 | hsa-miR-671-5p | -4.45 |
| hsa-miR-31-5p | -14.44 | hsa-miR-30a-5p | -11.48 | hsa-miR-532-5p | -4.02 |
| hsa-miR-574-3p | -10.54 | hsa-miR-370-3p | -8.38 | hsa-miR-140-3p | -3.81 |
| hsa-miR-125b-2-3p | -8.66 | hsa-miR-663a | -7.43 | **HSP90AA1** |  |
| hsa-miR-370-3p | -8.38 | hsa-miR-339-5p | -7.22 | hsa-miR-361-5p | -69.26 |
| hsa-miR-28-5p | -8.31 | hsa-miR-744-5p | -6.94 | hsa-miR-425-5p | -33.79 |
| hsa-miR-708-5p | -6.41 | hsa-miR-193b-5p | -6.85 | hsa-miR-16-5p | -7.98 |
| hsa-miR-30a-3p | -5.01 | hsa-miR-652-3p | -5.88 | hsa-let-7b-5p | -7.73 |
| hsa-miR-10b-3p | -3.7 | hsa-miR-339-3p | -5.8 | hsa-miR-30c-5p | -7.67 |
| hsa-miR-127-5p | -3.33 | hsa-miR-221-3p | -5.72 | hsa-miR-25-3p | -7.23 |
| hsa-miR-181a-3p | -3.14 | hsa-miR-381-3p | -5.67 | hsa-miR-491-5p | -6.22 |
| **ITGA2** |  | hsa-miR-874-3p | -5.31 | hsa-miR-139-5p | -5.65 |
| hsa-let-7a-5p | -31.63 | hsa-miR-324-3p | -5.2 | hsa-miR-324-3p | -5.2 |
| hsa-miR-22-3p | -30.84 | hsa-miR-362-5p | -5.1 | hsa-miR-362-5p | -5.1 |
| hsa-let-7d-5p | -29.9 | hsa-miR-671-5p | -4.45 | hsa-miR-337-5p | -4.85 |
| hsa-miR-103a-3p | -14.87 | hsa-miR-17-5p | -3.53 | hsa-miR-378a-3p | -4.14 |
| hsa-miR-195-5p | -14.04 | hsa-miR-24-3p | -3.53 | hsa-miR-10a-5p | -4.02 |
| hsa-miR-497-5p | -12.3 | hsa-miR-92b-5p | -3.26 | hsa-miR-140-3p | -3.81 |
| hsa-miR-107 | -11.99 | hsa-miR-23b-5p | -3.04 | hsa-miR-17-5p | -3.53 |
| hsa-miR-30a-5p | -11.48 | hsa-miR-378a-5p | -3 | hsa-miR-185-5p | -3.39 |
| hsa-miR-15a-5p | -10.66 | hsa-miR-498 | 2.05 | hsa-miR-127-5p | -3.33 |
| hsa-miR-574-3p | -10.54 | hsa-miR-483-5p | 2.56 | hsa-miR-181a-3p | -3.14 |
| hsa-miR-125a-5p | -9.69 | **NFKB1** |  | **IL8** |  |
| hsa-miR-370-3p | -8.38 | hsa-let-7a-5p | -31.63 | hsa-miR-146a-5p | -29.52 |
| hsa-miR-106b-5p | -8.08 | hsa-miR-146a-5p | -29.52 | hsa-miR-134-5p | -14.82 |
| hsa-miR-16-5p | -7.98 | hsa-miR-199a-5p | -11.9 | hsa-miR-23a-3p | -14.18 |
| hsa-miR-25-3p | -7.23 | hsa-miR-15a-5p | -10.66 | hsa-miR-451a | -10.45 |
| hsa-miR-130b-3p | -6.11 | hsa-miR-125a-5p | -9.69 | hsa-miR-93-5p | -4.15 |
| hsa-miR-652-3p | -5.88 | hsa-miR-146b-5p | -7.14 | hsa-miR-197-3p | -4.03 |
| hsa-miR-337-5p | -4.85 | hsa-miR-652-3p | -5.88 | hsa-miR-155-5p | -3.49 |
| hsa-miR-122-5p | -4.69 | hsa-miR-339-3p | -5.8 | **CXCL2** |  |
| hsa-miR-20a-5p | -4.67 | hsa-miR-139-5p | -5.65 | hsa-miR-27a-3p | -4.89 |
| hsa-miR-93-5p | -4.15 | hsa-miR-671-5p | -4.45 | hsa-miR-532-5p | -4.02 |
| hsa-miR-432-5p | -4.02 | hsa-miR-324-5p | -4.31 | hsa-miR-654-3p | -3.23 |
| hsa-miR-15b-5p | -3.98 | hsa-miR-532-5p | -4.02 | **PPBP** |  |
| hsa-miR-20b-5p | -3.72 | hsa-miR-15b-5p | -3.98 | hsa-miR-151a-3p | -32.1 |
| hsa-miR-17-5p | -3.53 | hsa-miR-155-5p | -3.49 | hsa-miR-146a-5p | -29.52 |
| hsa-miR-24-3p | -3.53 | **CHD3** |  | hsa-miR-31-5p | -14.44 |
| hsa-miR-106a-5p | -3.18 | hsa-miR-1273g-3p | -64.17 | hsa-miR-29a-3p | -8.8 |
| **CXCL6** |  | hsa-miR-409-3p | -28.06 | hsa-miR-146b-5p | -7.14 |
| hsa-miR-199a-5p | -11.9 | hsa-miR-150-5p | -23.41 | hsa-miR-27a-3p | -4.89 |
| hsa-miR-130a-3p | -8.11 | hsa-miR-103a-3p | -14.87 | hsa-miR-494-3p | -4.06 |
| hsa-miR-140-3p | -3.81 | hsa-miR-382-5p | -13.14 | hsa-miR-106b-3p | -3.33 |
| **SMAD4** |  | hsa-miR-107 | -11.99 | hsa-miR-452-5p | -3.06 |
| hsa-miR-145-5p | -55.42 | hsa-let-7e-5p | -11.7 | hsa-miR-483-5p | 2.56 |
| hsa-miR-146a-5p | -29.52 | hsa-miR-30a-5p | -11.48 | **SMAD3** |  |
| hsa-miR-26a-5p | -13.97 | hsa-miR-130a-3p | -8.11 | hsa-miR-145-5p | -55.42 |
| hsa-miR-199a-5p | -11.9 | hsa-miR-16-5p | -7.98 | hsa-miR-491-5p | -6.22 |
| hsa-miR-125a-5p | -9.69 | hsa-let-7b-5p | -7.73 | hsa-miR-143-3p | -4.17 |
| hsa-miR-130a-3p | -8.11 | hsa-miR-132-3p | -7.55 | **TP53** |  |
| hsa-miR-154-5p | -5.1 | hsa-miR-532-3p | -7.12 | hsa-miR-191-5p | -71.26 |
| hsa-miR-27a-3p | -4.89 | hsa-miR-744-5p | -6.94 | hsa-miR-361-5p | -69.26 |
| hsa-miR-452-5p | -3.06 | hsa-miR-130b-3p | -6.11 | hsa-miR-99b-5p | -59.57 |
| hsa-miR-498 | 2.05 | hsa-miR-337-5p | -4.85 | hsa-miR-145-5p | -55.42 |
| hsa-miR-4668-5p | 3.73 | hsa-miR-92b-5p | -3.26 | hsa-miR-22-3p | -30.84 |
| **JUND** |  | hsa-miR-654-3p | -3.23 | hsa-miR-150-5p | -23.41 |
| hsa-miR-125a-5p | -9.69 | hsa-miR-23b-5p | -3.04 | hsa-miR-30d-5p | -19.32 |
| hsa-miR-663a | -7.43 | hsa-miR-498 | 2.05 | hsa-miR-342-3p | -18.02 |
| hsa-miR-4707-5p | -4.31 | **CREBBP** |  | hsa-miR-486-5p | -15.04 |
| hsa-miR-494-3p | -4.06 | hsa-miR-100-5p | -51.6 | hsa-miR-134-5p | -14.82 |
| hsa-miR-139-3p | -3.09 | hsa-let-7e-5p | -11.7 | hsa-miR-26a-5p | -13.97 |
| hsa-miR-23b-5p | -3.04 | hsa-miR-130a-3p | -8.11 | hsa-miR-10b-5p | -12.27 |
| hsa-miR-1908-5p | 3.15 | hsa-miR-130b-3p | -6.11 | hsa-miR-574-3p | -10.54 |
| **EP300** |  | hsa-miR-324-3p | -5.2 | hsa-miR-151a-5p | -10.22 |
| hsa-miR-23b-3p | -35.32 | **USP12** |  | hsa-miR-29a-3p | -8.8 |
| hsa-miR-150-5p | -23.41 | hsa-let-7d-5p | -29.9 | hsa-miR-370-3p | -8.38 |
| hsa-miR-193b-3p | -20.35 | hsa-miR-409-3p | -28.06 | hsa-miR-214-5p | -8.1 |
| hsa-miR-574-3p | -10.54 | hsa-miR-195-5p | -14.04 | hsa-miR-193b-5p | -6.85 |
| hsa-miR-451a | -10.45 | hsa-miR-497-5p | -12.3 | hsa-miR-491-5p | -6.22 |
| hsa-let-7b-5p | -7.73 | hsa-miR-15a-5p | -10.66 | hsa-miR-24-2-5p | -5.92 |
| hsa-miR-30c-5p | -7.67 | hsa-miR-130a-3p | -8.11 | hsa-miR-339-3p | -5.8 |
| hsa-miR-25-3p | -7.23 | hsa-miR-16-5p | -7.98 | hsa-miR-381-3p | -5.67 |
| hsa-miR-30a-3p | -5.01 | hsa-let-7b-5p | -7.73 | hsa-miR-139-5p | -5.65 |
| hsa-miR-337-5p | -4.85 | hsa-miR-132-3p | -7.55 | hsa-miR-21-3p | -5.23 |
| hsa-miR-4758-5p | -3.35 | hsa-miR-339-3p | -5.8 | hsa-miR-6722-3p | -5.15 |
| hsa-miR-181b-5p | -3.09 | hsa-miR-15b-5p | -3.98 | hsa-miR-1233-5p | -5.09 |
| hsa-miR-4745-5p | 2.86 | hsa-miR-409-5p | -3.93 | hsa-miR-337-5p | -4.85 |
| hsa-miR-4481 | 3.01 | hsa-miR-498 | 2.05 | hsa-miR-671-5p | -4.45 |
| **SMARCA4** |  | **OSBPL10** |  | hsa-miR-4651 | -4.41 |
| hsa-miR-21-5p | -10.11 | hsa-miR-21-5p | -10.11 | hsa-miR-2110 | -4.28 |
| hsa-miR-708-5p | -6.41 | hsa-miR-708-5p | -6.41 | hsa-miR-143-3p | -4.17 |
| hsa-miR-27a-3p | -4.89 | hsa-miR-27a-3p | -4.89 | hsa-miR-532-5p | -4.02 |
| hsa-miR-6779-5p | -4.78 | hsa-miR-6779-5p | -4.78 | hsa-miR-664a-5p | -3.89 |
| hsa-miR-122-5p | -4.69 | hsa-miR-122-5p | -4.69 | hsa-miR-1909-3p | -3.61 |
| hsa-miR-7977 | -4.67 | hsa-miR-7977 | -4.67 | hsa-miR-377-5p | -3.56 |
| hsa-miR-143-5p | -3.9 | hsa-miR-143-5p | -3.9 | hsa-miR-185-5p | -3.39 |
| hsa-miR-24-3p | -3.53 | hsa-miR-24-3p | -3.53 | hsa-miR-106b-3p | -3.33 |
| hsa-miR-155-5p | -3.49 | hsa-miR-155-5p | -3.49 | hsa-miR-629-5p | -3.12 |
| hsa-miR-6769b-5p | 2.29 | **ERG** |  | hsa-miR-139-3p | -3.09 |
| hsa-miR-483-5p | 2.56 | hsa-miR-99b-5p | -59.57 | hsa-miR-452-5p | -3.06 |
| hsa-miR-297 | 3.81 | hsa-miR-145-5p | -55.42 | hsa-miR-937-5p | -3.03 |
| **MAG** |  | hsa-miR-100-5p | -51.6 | hsa-miR-7110-5p | 2.13 |
| hsa-let-7a-5p | -31.63 | hsa-miR-151a-3p | -32.1 | hsa-miR-6127 | 2.73 |
| hsa-let-7d-5p | -29.9 | hsa-miR-382-5p | -13.14 | hsa-miR-7150 | 3.86 |
| hsa-miR-193b-3p | -20.35 | hsa-miR-30a-5p | -11.48 | hsa-miR-8071 | 5.14 |
| hsa-let-7i-5p | -8.38 | hsa-miR-193a-5p | -10.93 | **TIMP3** |  |
| hsa-miR-28-5p | -8.31 | hsa-miR-15a-5p | -10.66 | hsa-miR-17-3p | -4.6 |
| hsa-miR-339-3p | -5.8 | hsa-miR-151a-5p | -10.22 | **A2M** |  |
| hsa-miR-874-3p | -5.31 | hsa-miR-106b-5p | -8.08 | hsa-miR-151a-3p | -32.1 |
| hsa-miR-324-3p | -5.2 | hsa-let-7b-5p | -7.73 | hsa-miR-362-5p | -5.1 |
| hsa-miR-342-5p | -4.46 | hsa-miR-25-3p | -7.23 | hsa-miR-122-5p | -4.69 |
| hsa-miR-483-5p | 2.56 | hsa-miR-362-5p | -5.1 | **PZP** |  |
| hsa-miR-6127 | 2.73 | hsa-miR-665 | -5.1 | hsa-miR-134-5p | -14.82 |
| **TIMP4** |  | hsa-miR-20a-5p | -4.67 | hsa-miR-455-3p | -3.45 |
| hsa-miR-151a-3p | -32.1 | hsa-miR-17-3p | -4.6 | **CAPN1** |  |
| hsa-miR-146a-5p | -29.52 | hsa-miR-671-5p | -4.45 | hsa-miR-17-3p | -4.6 |
| hsa-miR-146b-5p | -7.14 | hsa-miR-4507 | -4.42 | **SHBG** |  |
| hsa-miR-193b-5p | -6.85 | hsa-miR-324-5p | -4.31 | hsa-miR-25-3p | -7.23 |
| hsa-miR-337-5p | -4.85 | hsa-miR-151b | -4.2 | hsa-miR-193b-5p | -6.85 |
| hsa-miR-532-5p | -4.02 | hsa-miR-93-5p | -4.15 | hsa-miR-10a-5p | -4.02 |
| **ITGB1** |  | hsa-miR-532-5p | -4.02 | hsa-miR-654-3p | -3.23 |
| hsa-miR-145-5p | -55.42 | hsa-miR-143-5p | -3.9 | **LIG4** |  |
| hsa-miR-103a-3p | -14.87 | hsa-miR-17-5p | -3.53 | hsa-miR-26a-5p | -13.97 |
| hsa-miR-134-5p | -14.82 | hsa-miR-24-3p | -3.53 | hsa-miR-16-5p | -7.98 |
| hsa-miR-23a-3p | -14.18 | hsa-miR-155-5p | -3.49 | hsa-miR-132-3p | -7.55 |
| hsa-miR-497-5p | -12.3 | hsa-miR-92b-3p | -3.43 | hsa-miR-155-5p | -3.49 |
| hsa-miR-107 | -11.99 | hsa-miR-106b-3p | -3.33 | hsa-miR-181a-3p | -3.14 |
| hsa-miR-21-5p | -10.11 | hsa-miR-654-3p | -3.23 | **PAN2** |  |
| hsa-miR-125a-5p | -9.69 | hsa-miR-1307-3p | -3.2 | hsa-miR-29a-3p | -8.8 |
| hsa-miR-370-3p | -8.38 | hsa-miR-106a-5p | -3.18 | hsa-miR-487b-3p | -5.06 |
| hsa-miR-106b-5p | -8.08 | hsa-miR-181a-3p | -3.14 | hsa-miR-377-5p | -3.56 |
| hsa-miR-25-3p | -7.23 | hsa-miR-629-5p | -3.12 | hsa-miR-297 | 3.81 |
| hsa-miR-193a-3p | -7.13 | hsa-miR-452-5p | -3.06 | **HNF1A** |  |
| hsa-miR-130b-3p | -6.11 | hsa-miR-498 | 2.05 | hsa-miR-15b-5p | -3.98 |
| hsa-miR-339-3p | -5.8 | hsa-miR-297 | 3.81 | **ITGA5** |  |
| hsa-miR-221-3p | -5.72 | **KIAA0101** |  | hsa-miR-31-5p | -14.44 |
| hsa-miR-381-3p | -5.67 | hsa-miR-1273g-3p | -64.17 | hsa-miR-382-5p | -13.14 |
| hsa-miR-139-5p | -5.65 | hsa-miR-181a-5p | -21.34 | hsa-miR-25-3p | -7.23 |
| hsa-miR-154-5p | -5.1 | hsa-miR-193b-3p | -20.35 | hsa-miR-708-5p | -6.41 |
| hsa-miR-487b-3p | -5.06 | hsa-miR-451a | -10.45 | hsa-miR-30a-3p | -5.01 |
| hsa-miR-20a-5p | -4.67 | hsa-miR-106b-5p | -8.08 | hsa-miR-432-5p | -4.02 |
| hsa-miR-17-3p | -4.6 | hsa-miR-130b-3p | -6.11 | hsa-miR-92b-3p | -3.43 |
| hsa-miR-342-5p | -4.46 | hsa-miR-652-3p | -5.88 | **DDR1** |  |
| hsa-miR-324-5p | -4.31 | hsa-miR-21-3p | -5.23 | hsa-miR-199a-5p | -11.9 |
| hsa-miR-93-5p | -4.15 | hsa-miR-122-5p | -4.69 | hsa-miR-663a | -7.43 |
| hsa-miR-409-5p | -3.93 | hsa-miR-20a-5p | -4.67 | hsa-miR-744-5p | -6.94 |
| hsa-miR-140-3p | -3.81 | hsa-miR-664b-3p | -4.19 | hsa-miR-342-5p | -4.46 |
| hsa-miR-20b-5p | -3.72 | hsa-miR-93-5p | -4.15 |  |  |
| hsa-miR-17-5p | -3.53 | hsa-miR-433-3p | -3.89 |  |  |
| hsa-miR-24-3p | -3.53 | hsa-miR-379-5p | -3.88 |  |  |
| hsa-miR-455-3p | -3.45 | hsa-miR-20b-5p | -3.72 |  |  |
| hsa-miR-127-5p | -3.33 | hsa-miR-17-5p | -3.53 |  |  |
| hsa-miR-106a-5p | -3.18 | hsa-miR-24-3p | -3.53 |  |  |
| hsa-miR-139-3p | -3.09 | hsa-miR-106a-5p | -3.18 |  |  |
| hsa-miR-6126 | -3 |  |  |  |  |

**S2 Table**. 1001 miRNAs associated with the regulation of 81 genes connected with COL1A2, COL3A1, MMP9 and MMP2.
